# Rheumatism in Its Professional and Popular Aspects

**Published:** 1915-10-30

**Authors:** 


### RHEUMATISM IN ITS PROFESSIONAL AND POPULAR ASPECTS.

Of making of books and papers on rheumatism there is apparently no end. The name must be one of the oldest in medicine, and one might have thought that all that could be said on the subject had long since been exhausted. Yet even to the present moment the interest appears to continue, and journals—which presumably know the wants of their readers—hopefully promote the discussion. Quite recently one of our American contemporaries has issued a large special number in which some thirty writers address themselves to an exposition of the various features of the disease, and there are also abundant evidences of continuing interest nearer home. To say that all this writing and contributing means a corresponding measure of advance either in knowledge or in practice would hardly be affirmed even by the most flexible critic. Indeed, even minute evidence of any such movement is hard to detect. Still, the new minds constantly arriving in medicine have to be considered, and the journals of the day and hour must remember these as well as those who have long stories of personal observation and reading to their credit. Even if our writers do not give us something new and original, they have the opportunity of setting forth what is known in a lucid manner, and at the very least they may remind us of something we have managed to forget. We cannot say they are always successful in the former of these ambitions, though the latter is readily within their reach. Perhaps, to judge from recent examples, a little more credit might be given to the original sources of inspiration. Thus one would have thought it an impossible task to write about rheumatism in childhood without mention of the name of the late Dr. Cheadle, and yet some of our modern instructors have achieved this result. Indeed, one contributor, addressing the Registered Medical Women's Association, announces that what she knows on the subject has been almost entirely self-taught, for "there is not much to be learnt about it from current literature." Apparently she has never even heard of a small volume published in 1889 and entitled "Lectures on the Rheumatic State in Childhood," for she started work in 1904 "believing that there was very little rheumatism in children." If we may venture another general criticism it would be that many writers seem inclined to tell us what they "believe" or "think," while failing to produce the evidence on which their convictions are based. Now, while the personal creed of a writer may be a matter of domestic interest, and in time even of historical interest, it is of little concern to his contemporaries, many of whom can have no knowledge of his personal values, and all of whom want evidences and not dogmas. Our suggestion is that of these there is now enough and to spare, and that the future will be content with facts and with arguments based on facts.

### What is "Rheumatism"?

One difficulty in attempting to set forth the modern view of rheumatic disease is that not all the contributors use the term in one and the same sense. Everyone, or nearly everyone, is agreed that there is an acute febrile disease distinguished by pain and swelling of the joints, by free sweating, and by some risk of valvular or other disease of the heart; and that this disease may be called acute rheumatism or rheumatic fever. What is the essential cause of this is a matter of uncertainty. Some affirm that the cause is a specific micro-organism, while others hold that there is no sufficient evidence for this conclusion. Again, there is general agreement that acute rheumatism, not uncommon in the adult, is even more frequent in childhood, but with a very different clinical picture. Here pain and swelling of the joints are usually inconspicuous or absent, while the more prominent features may be such events as tonsillitis, sore throat, growing pains, St. Vitus's dance, certain skin eruptions, subcutaneous nodules, etc. Indeed, it may be said that in children of a rheumatic inheritance the disease shows itself in so many ways that one can hardly exclude any event and affirm confidently "This, at least, is not rheumatism." Now, this is the more important for two reasons. First, it is certain that in any active display of rheumatism in childhood there is a considerable risk that the heart may become involved; and secondly, as in the popular view rheumatism is a disease of the joints, the non-arthritic manifestations of the disease as seen in childhood do not come under this term in the lay vocabulary. Every doctor again and again has seen an unhappy child with manifest signs of heart disease, though the mother protests in all good faith that the child has never had "rheumatism." The explanation in the great majority of cases is that the heart was affected during some expression of the disease such as those above-mentioned; and not infrequently the attack is such a slight affair that little attention is paid to it either at the time or afterwards. This is repeatedly the melancholy story of heart disease in early life. Rheumatism in a non-arthritic form, and therefore in a non-recognised form, has occurred, and during the active phase of the disease the structures of the heart have been attacked. It is easy to see how this occurrence is especially likely to arrive among the poorer classes of the community, where what appear to be slight ailments in the children are apt to be overlooked; and as a fact it is at this social level that heart disease is more frequent. The only chance of prevention is to forewarn parents in families in which there is known to be a rheumatic strain. If it were a universal professional rule to point out to the parents whenever a rheumatic tendency is discovered the risks which the apparently slight illnesses of their children involve, something would be gained. The parents would be made alive to the real situation, and they would thus recognise the special need for care even when their children's symptoms were not obviously severe and were not attended by pains in the joints.

It may be asked, How would this practice prevent heart disease? The answer is that it would help to secure early and complete rest during the non-arthritic manifestations of the disease. Admittedly this is the influence which is most likely to prevent cardiac complications in rheumatism. It is practised by the adult because his painful joints drive him to it whether he will or no. The child readily avoids it because his rheumatism is generally non-arthritic, is concealed under other names, and is often a relatively slight affair. In the adult rheumatic heart disease (except as persisting from early life) is comparatively uncommon; in the child it is frequent. Hence the suggestion that by education of the parents in rheumatic families there is some chance of diminishing the incidence of heart disease in childhood, seeing that this incidence is associated with the occurrence of rheumatism in forms which do not receive lay recognition, and which often fail to receive the only available means of protection—namely, early and complete rest. How far this method would be successful no one can pretend to say. But if rest is admitted to have some preventive value, an attempt ought to be made to secure it. No other method than the one here proposed has been suggested, and even if its promise is but a limited one it ought, none the less, to be applied for what it is worth.

### Chronic Joint Troubles.

Up to this point there is substantial agreement in the medical profession, and in so far as there is a conflict with public habit the conflict is an important one, as is above suggested. The education of the public, it is here suggested, might lessen the number of victims of rheumatic heart disease. But when we pass from more or less acute manifestations to those which are chronic in their course, difference of opinion in reference to the application of the terms "rheumatism" and "rheumatic" abound. Take the great group of cases distinguished by gradually increasing thickenings of joints and producing deformities more particularly of the hands and feet. Such names as rheumatoid arthritis, osteo-arthritis, and rheumatic gout are commonly applied to them, but regarding their true causation there is no certainty. Toxins, bacteria, reflex irritation have all been accused, but proof of every one of these is wanting. Arthritis due to gout may be recognised, and also that due to urethritis and the variety following certain diseases of the spinal cord. But outside these there is a great group of cases in which the course is chronic and apt to be progressive, and to which, as the cause is unknown, it is hardly justifiable to apply any more distinctive name than chronic arthritis or chronic polyarthritis. Until comparatively recent years these cases were commonly regarded, even in the medical profession, as examples of chronic rheumatism, on the view, presumably, that they were the consequence of the same agency, whatever this may be, which produces rheumatic fever; and even yet to the public there is a chronic rheumatism which appears in the form of joint disease. The present general tendency, both within and without the profession, is to question this suggestion, but obviously, so long as the cause of these chronic conditions is unknown, we cannot confidently say that it is certainly not identical with the equally unknown cause of rheumatic fever.

### A Question of Nomenclature.

This latter assumption, however, may be challenged, and indeed, it has been challenged, and this, too, over a wider area than the one just presented. Rheumatic fever, it has been said, is something quite apart from "rheumatics" or "rheumatism," and history may be summoned to defend the thesis. There is no doubt that in its original use the word "rheumatism" had no relation to diseases of the joints. It was used to signify conditions attended with a "rheum" or mucous discharge such as would nowadays be called a catarrh, and this meaning is preserved in the French words *le rhume* and *enrhumer*. How some two hundred and fifty years ago the term began to be applied to joint disturbances is uncertain, but the custom has had a strong hold in this country. Perhaps the most ingenious suggestion for reconciling the original use of the word with more modern practice is that proposed by the late Sir Jonathan Hutchinson, who defined a rheumatic person as one "prone to catarrhal cold in his joints"—that is, as the disease was characterised by fluid effusion into the joints this effusion might be named a "rheum" and the condition in which this occurred was termed "rheumatism." Whatever be the real explanation, it is easy to see that when painful swellings of the joints were once called rheumatism all such painful swellings would be apt to be so described, and, indeed, for long even gout was included under this name. Further, from painful joints the term would easily spread to pains in the neighbourhood of joints, and thus the term "muscular rheumatism" would in due time appear. Hence, even at the present day, the public habit is to call all pains which have no obvious causation "rheumatism," at least until the sufferer meets with someone who insists that the condition is "neuritis." From the point of view of nomenclature it is thus evident that the question is a much entangled one, and there is little chance of producing a clearly defined plan until the causation of the various conditions more or less commonly named "rheumatic" is discovered. Rheumatic fever we know, and the rheumatic manifestations of early life with their special risk of heart disease. But whether beyond these there are affections of the joints and other tissues which are merely the same disease in a chronic form, is entirely an open question.

